# The 2015 Outbreak of Severe Influenza in Kashmir, North India: Emergence of a New Clade of A/H1n1 Influenza Virus

**DOI:** 10.1371/currents.outbreaks.519e170f2740fabd4ccd1642ff533364

**Published:** 2018-08-08

**Authors:** Parvaiz Koul, Varsha Potdar, Hyder Mir, Mandeep Chadha

**Affiliations:** Internal & Pulmonary Medicine, Sheri Kashmir Institute of Medical Sciences, Srinagar, J&K, India; National Institute of Virology, Pune, India; Internal & Pulmonary Medicine, Sheri Kashmir Institute of Medical Sciences, Srinagar, J&K, India; National Institute of Virology, Pune, India

**Keywords:** emerging infectious diseases, infectious disease, Influenza

## Abstract

Introduction: Following the initial outbreak of A/H1N1pdm09, periodic resurgences of the virus, with variable morbidity and mortality, have been reported from various parts of India including the temperate Kashmir region of northern India. An outbreak of A/H1N1 was reported in early 2015 across India with a high morbidity and mortality. We studied patients during the outbreak in Kashmir.

Methods: Patients (n=1780, age 1 month to 90 years, median 35 years) presenting with acute respiratory illness to a tertiary care hospital in Srinagar, Kashmir from October 2014 to April 2015 were recruited. After clinical data recording, combined throat and nasal swabs were collected in viral transport medium and tested by real-time RT-PCR for influenza viruses. All influenza A positive samples were further subtyped using primers and probes for A/H1N1pdm09 and A/H3 whereas influenza B samples were further subtyped into B/Yamagata and B/Victoria lineages. Virus isolation, hemagglutination inhibition testing, sequencing and phylogenetic analysis was carried out using standard procedures. Testing for H275Y mutation was done to determine sensitivity to oseltamivir. All patients received symptomatic therapy and influenza positive patients were administered oseltamivir.

Results: Of the 1780 patients, 540 (30%) required hospitalization and 533 tested positive for influenza [influenza A=517(A/H1N1pdm09=437, A/H3N2=78 with co-infection of both in 2 cases); influenza B=16 (B/Yamgata=15)]. About 14% (n=254) had been vaccinated against influenza, having received the NH 2014-15 vaccine, 27 (11.3%) of these testing positive for influenza.  Sixteen patients, including 4 pregnant females, died due to multi-organ failure. HA sequencing depicted that 2015 isolates belonged to Clade 6B.1. No H275Y mutation was reported from A/H1N1 positives.

Conclusion: Resurgent outbreak of A/H1N1pdm09, with emergence of clade 6B.1, in 2014-15 resulted in high rate of hospitalizations, morbidity and mortality. Periodic resurgences and appearance of mutants emphasize continued surveillance so as to identify newer mutations with potential for outbreaks and severe outcomes.

## Introduction

Following the initial outbreak of A/H1N1pdm09 in 2009-10, periodic resurgences of the pandemic influenza virus have been reported from India with variable morbidity and mortality. 1^, ^2 We have earlier reported a recrudescence of A/H1N1pdm09 in 2012-13 in Kashmir region of the northern Indian state of Jammu and Kashmir where influenza is an important cause of acute respiratory infections during the winter months. 3^, ^4

During the initial months of 2015, an unusual increase in influenza A/H1N1pdm09 activity was observed thoughout India with more than 39000 cases and about 3000 deaths. 5 While the activity was usual for the temperate northern state of Jammu and Kashmir, it was unusual for the rest of the country which along with a more tropical geography traditionally witnesses high activity during summers coinciding with the rains of the monsoons. 1 Two scientists from the Massachusetts Institute of Technology, USA in an in silico analysis of retrieved sequences of 2014 from Genbank reported that the higher virulence of the A/H1N1pdm09 in 2015 was attributable to K166Q, D225N and T200A mutations in the HA region of the amino acid sequences in the receptor binding site of A/H1N1pdm09.^6^ More recently, studies from central, 7 and Eastern India,^8^ have reported a drift in the A/H1N1pdm09 virus and emergence of different clade of the virus which became the dominant circulating strain in consonance with similar trends across the globe.

We herewith report on the 2015 outbreak in the northern Indian state of Jammu and Kashmir which was associated with high morbidity and mortality and document a change in the genetic constitution of the virus that has implications on the vaccine strain for protection against influenza.

## Methods

Kashmir is the major province of the northern most Indian state of Jammu and Kashmir that borders China, Pakistan and Afghanistan. As against the rest of the country with more tropical climate, the valley of Kashmir bound by the Himalayas has a temperate geography with respiratory tract illnesses predominating during the winter months, much like the northern hemispherical seasonality of respiratory viral illnesses seen in Northern America and Western Europe. We have earlier documented pandemic and seasonal influenza viruses as a cause of respiratory illness in Kashmir,^3^^, ^4 that constitute the majority of hospital visits during the winter months, either as acute respiratory infections or as infective exacerbations of underlying chronic lung diseases like COPD.^9^ Sheri-Kashmir Institute of Medical Sciences (SKIMS) is a 820-bed facility in the summer capital, Srinagar, and constitutes the main tertiary referral centre for respiratory cases for the area. During the winter of 2014-2015, SKIMS witnessed an increase in hospital visits by patients with acute respiratory illness, many of whom required hospitalization. We performed surveillance for outpatients with acute respiratory infection (ARI), influenza-like illness (ILI) and in-patients with severe acute respiratory illness (SARI). Influenza like illness (ILI) was defined as fever of 100^0^F (>37.2^0^C) accompanied by cough and/or sore throat, whereas SARI was defined as those patients with ILI who also require hospitalization. Patients without fever were labeled as ARI.

All patients were interviewed for details of the illness and examined. Clinical history was specifically recorded for any history of contact with a case of ARI or proven influenza and any history of clustering (two or more cases that were related in time and space, e.g., in a home or workplace). After recording of the clinical data, combined throat and nasal swabs were collected in viral transport medium, transported to the influenza laboratory and tested by real-time RT-PCR for influenza viruses using the CDC protocol. 10 Influenza A positive samples were further subtyped using primers and probes for A/H1N1pdm09 and A/H3 and Influenza B positives were subtyped into B/Yamagata and B/Victoria lineages. Virus isolation, haemaggglutination inhibition testing, sequencing and phylogenetic analysis was carried out using standard assay procedures as described previously. 11^, ^12 Samples were also tested for NA mutations that could result in neuraminidase resistance.

Patients requiring hospitalization were admitted and all patients received symptomatic therapy and influenza positive patients were administered oseltamivir in addition to routine measures that included respiratory support by mechanical ventilation. Post mortem cesarean section was conducted in one deceased pregnant lady for delivering the 34-week fetus that survived after immediate neonatal care for 2 weeks.

In addition to the study samples, at the National Institute of Virology, Pune a total of 285 Indian HA sequences of the period 2009-2015 were rechecked for the 3 mutations reported by Tharakaraman and Sasisekharan. 6 Statistical analysis of all data was done using STATA 11 software using Fisher’s exact test and Chi-square for categorical variables and Student’s t-test for continuous variables. A p value of p<0.05 was considered significant.

**Ethics Statement:** The study was approved by the Institute Ethics Committee of SKIMS and informed consent for participation was obtained for all patients.

## Results

**Figure 1.**
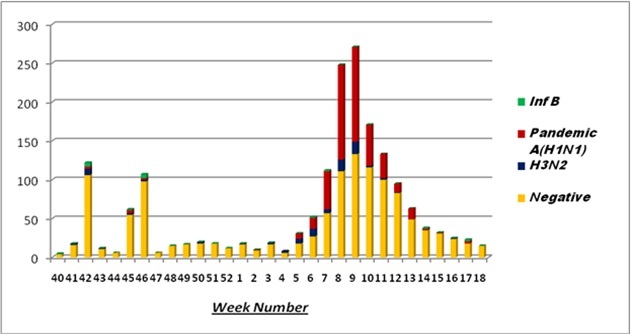
Weekwise positivity of influenza cases (October 2014-March 2015)

The 1780 recruited patients (845 male; with age 1 month to 90 years (median 35 years) presented with respiratory symptoms of varying severity, 540 (30%) required hospitalisation. The various symptoms experienced by the patients are depicted in table 1 and included respiratory symptoms and fever as the predominant manifestations. The respiratory samples tested positive for influenza in 533 (30%) cases. Table 1 also depicts the distribution of clinical features among influenza positive and influenza negative patients. The median duration of symptoms was 3 days in influenza positive patients compared to a median of 4 days among the influenza negative patients. Influenza positive patients were significantly more likely to have fever, cough, nasal discharge, body aches, fatigue and a history of an acute respiratory tract infection in the family (table 1). Most of the patients presented in the early weeks of 2015 (Figure 1), conforming to the previously documented seasonality of influenza in Kashmir.

**Table 1:** Demographic and clinical features of influenza-positive and influenza-negative patients.

	Influenza positive N (%)	Influenza negative N (%)	p-value
Number	533 (100)	1247 (100)	
Males	262 (49.1)	583 (46.7)	0.35
Age (Mean ± SD)	30.6 ± 19	38 ± 20.5	<0.0001
Duration of symptoms in days (median, range)	3 (1-25)	4 (1-30)	
Clinical features			
Fever	506 (94.9)	999 (80.1)	<0.0001
Cough	503 (94.3)	1098 (88.0)	<0.0001
Chills	451 (84.6)	842 (67.5)	<0.0001
Nasal discharge	411 (77.1)	956 (76.6)	0.84
Ear discharge	8 (1.5)	22 (1.7)	0.68
Sore throat	388 (72.7)	851 (68.2)	0.05
Breathlessness	351 (65.8)	771 (61.8)	0.10
Expectoration	231 (43.3)	549 (44.0)	0.79
Headache	382 (71.6)	829 (66.4)	0.03
Body ache	407 (76.3)	847 (67.9)	0.003
Fatigue	404 (75.7)	803 (64.3)	<0.0001
Concomitant illness	23 (4.3)	121 (9.7)	0.0001
ARI in the family	151 (28.3)	186 (14.9)	<0.0001
Vomiting	113 (21.2)	218 (17.4)	0.06
Diarrhea	64 (12.0)	110 (8.8)	0.038
Seizures	8 (1.5)	19 (1.5)	1.0
Vaccination status			
Vaccinated	27 (5.0)	227 (18.2)	<0.0001

**Figure 2.**
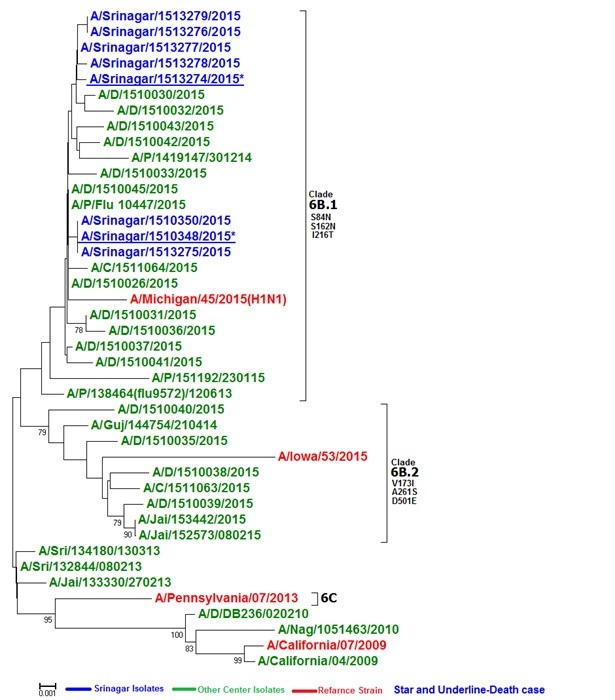
HA phylogenetic analysis of 2015 isolates of A/H1N1pdm09 . The tree consists of 2015 isolates from Pune, Delhi and compared with Srinagar isolates .The asterix and underline isolates are from the fatal severe cases . The 2015-16 vaccine component is shown in red font. Srinagar strains are in blue font. 2015 isolates belong to Clade 6B.1 with clade specific S203T or A203T, and D97N signature mutations

The influenza viruses that were detected in the 533 influenza-positive included 517 cases with influenza A (A/H1N1pdm09=437, A/H3N2=78 with 2 co-infected with both) and 16 cases with influenza B ( B/Yamagata=15)]. HA sequencing and phylogenetic analysis of the A/H1N1 sequences depicted that 2015 isolates from Kashmir clustered with clade 6B.1 with clade specific S84N, S162N and I216T signature mutations (Figure 2). HA-sequencing did not demonstrate any evidence of K166Q, D225 or T200A mutation, even in those who had a fatal outcome of their infection. No H275Y mutation on neuraminidase was reported from pandemic H1N1 positives, hence viruses remained susceptible to oseltamivir.

All influenza positive patients received oral oseltamivir, 16 patients died due to multiorgan failure whereas the rest had an uncomplicated course with full recovery. The patients who died included 4 females who had developed influenza during their pregnancy.^13^

A rechecking of the analysis of the 285 Indian HA sequences of the period 2009-2015 was performed and it was found that K166Q mutation was established in Indian strains from 2013 (Table 2). In addition at position 200, the residue has been ‘A’ itself from the beginning strains of 2009. In 2012 few Indian strains were observed with A200T mutation which did not get established and further it continued as A200 since 2013.

**Table 2:**
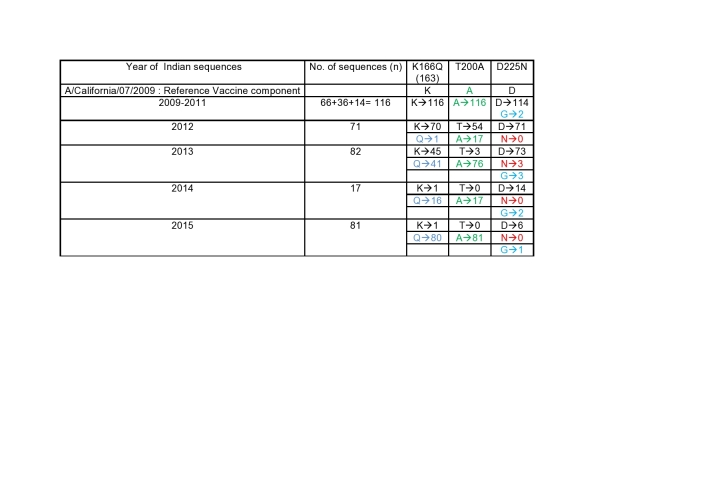
Details of the HA sequences of the strains over the years.

## Discussion

Our data documents a virulent outbreak of influenza caused predominantly by A/H1N1 virus which upon genotyping belonged to clade 6 as against the classical clade 7 of the A/California09 virus, with few signature mutations associated with the genotype 6.1. There was a high morbidity of the illness with about a third of the patients requiring admission and about 16 influenza-related deaths, including 4 pregnant patients reported earlier. 13 The heightened activity in Kashmir coincided with similar outbreaks in the rest of the country, where previous studies have demonstrated a seasonality coinciding with the summer months and with monsoon rains, 1^, ^2 even as geographically the country as a whole is geographically located in the northern hemisphere.

Following the emergence of A/H1N1pdm in 2009, the strain largely replaced the circulating seasonal influenza A/H1N1) and Inf A/H3N2 viruses and from 2010 onwards continued to circulate replacing the previously circulating seasonal A/H1N1 along with A/H3 and influenza B. The circulation of these viruses continued with seasonal activity with resurgence of A/H1N1pdm09 in several Indian states in 2012-2013 including the northern state of Jammu & Kashmir.^2^ ^14^

Since 2013, several reports have indicated the emergence of an expanding clade of A/H1N1pdm09 viruses, designated 6B. 7^, ^15^, ^16 This subgroup appeared in 2012-13 and became predominant in 2013-14. According the World Health Organization, antigenic characteristics of A/H1N1pdm09 viruses collected globally from September 2015 to January 2016 indicated that almost all the A/H1N1pdm09 viruses were antigenically similar and closely related to the vaccine virus A/California/7/2009. These included severe and fatal cases as well. However the sequencing of the HA genes of these viruses indicated the emergence of two new sucbclades within the subgroup 6B (6B.1 and 6B.2) of these A/H1N1pdm09 viruses. The relative proportion of clade 6B viruses expanded from late 2015 and 6B.1 became predominant in most of the geographies except China where the subclade 6B.2 predominated. Our data is in consonance with the global trend observed by the WHO. Pertinently, while the viruses within these subclades are not antigenically distinguishable from A/California/7/2009-like viruses, some recent reports indicate that A/H1N1pdm09 viruses within the 6B.1 and 6B.2 subclades reacted poorly with sera from individuals vaccinated with A/California/7/2009-like-strain-containing vaccine. 17

The first report of genotype 6B strains circulating in India reported by Parida et al^7^ who demonstrated genotype 6B forming two sub-lineages circulated during the outbreak in Madhya Pradesh in central India harbouring the signature amino acid substitutions of genogroup 6B (D97N, K163Q, S185T, S203T, A256T and K283E). They also noted a new mutation E164G in HA2 sequences. A subsequent study from Eastern India also demonstrated the clustering of the viruses with globally circulating clade 6B. The D225N mutation reported by MIT investigators was not demonstrated but T200A was found to be conserved.^8^ Our study shows the emergence of subclades of 6B, 6B.1 and 6B.2 in Kashmir, the former predominating.

With a high morbidity and mortality of the 2015 outbreak, it was widely believed that the A/H1N1 virus had mutated and thus rendered more virulent and potentially lethal. The investigators from the Massachusetts Institute of Technology, on the basis of an in-silico analysis of the Genbank submitted Indian-origin strain A/India/6427/2014 reported amino acid T200A and D225N changes that were different from the original A/H1N1pdm09 strain.^6^ The T200A aminoacid substitution ensures an enhanced human glycan receptor binding of the HA antigen of the influenza virus^18^ and the D225N substitution leads to increased virulence and disease severity. 19 The D225N mutation has also been reported to affect receptor binding of the HA whereas it also results in reduced susceptibility to neuraminidase (NA), 20 and has previously also been reported to be associated with serious influenza illness requiring hospitalisation or death. 21 The authors believed that the chance of person to person transmission in India with high population density created opportunities for the strain to sustain and they become dominant. The set of mutations that they reported to characterise the strain were K166Q, T200A, and D225N. They also found some N129, G158, and N159 as other important HA changes observed in the retrieved 2014 Indian-origin sequences. Our data along with the analysis of the 285 Indian HA sequences showed that the K166Q mutation was established in Indian strains since 2013. In addition at position 200 the residue has been `A’ itself since the beginning. In 2012 few Indian strains were observed with A200T mutation which did not get established and further it continued as A200 since 2013. The D225N mutation has not been observed in our isolates (including the fatal cases) or other Indian strains with the exception of 3 of 289 Indian strains isolated form severe as well as non severe cases of the year 2013 from Pune. Our isolates clustered with clade 6B.1 and 6B.2. Phylogenetic analysis of other strains from Delhi and Pune also depicted most of the isolates clustering with subclades of clade 6B. Since the circulating strain was drifted from the vaccine strain, it could have resulted in a poorer efficacy of the vaccine in 2014-15. WHO, CDC and the ICMR have now recommended a change of the A/California 09 to the A/Michigan/2015like virus which is likely to have a better match with the circulating A/H1N1virus.

Another important observation in our study was the absence of the H275Y mutation on neuraminidase in our isolates which suggested that the isolates were susceptible to oseltamivir. This has significant implications from the treatment perspective of the patients and as such oseltamivir can continue to be used as per the guidelines.

In conclusion our data demonstrates that A/H1N1 continues to evolve and in the outbreak of 2015 in Kashmir, there was emergence of clade 6B.1 of A/H1N1 which continues to circulate as the dominant strain of seasonal A/H1N1 influenza. Our data emphasize the importance of continued surveillance from wider areas of the country to keep tracking any antigenic changes that would dictate change in the vaccine strain for influenza infection.

## Competing Interests

The authors have declared that no competing interests exist.

## Data Availability Statement

The data has been uploaded into the public repository, with the DOI as: 10.6084/m9.figshare.5977717.
